# Baseline plasma-informed circulating tumor DNA analyses comparing multiplex digital PCR and NGS for longitudinal monitoring in Hodgkin lymphoma

**DOI:** 10.1038/s41408-026-01555-2

**Published:** 2026-06-27

**Authors:** Zahra Haider, Linn Deleskog Spångberg, Karin E. Smedby, Olha Krynina, Cecilia Jylhä, Irina Savitcheva, Emil Lundin, Marzia Palma, Lotta Hansson, Leonie Saft, Blaž Oder, Anna Lyander, Moa Hägglund, Anna Gellerbring, Mathias Johansson, Karl Nyrén, Richard Rosenquist, Tove Wästerlid, Emma Tham

**Affiliations:** 1 Department of Molecular Medicine and Surgery, Karolinska Institutet, Stockholm, Sweden; 2 Department of Neuropathology, Heidelberg University Hospital, Heidelberg, Germany; 3 Division of Clinical Epidemiology, Department of Medicine Solna, Karolinska Institutet, Stockholm, Sweden; 4 Department of Hematology, Karolinska University Hospital, Stockholm, Sweden; 5 Clinical Genetics and Genomics, Karolinska University Hospital, Stockholm, Sweden; 6 Medical Radiation Physics and Nuclear Medicine, Karolinska University Hospital, Stockholm, Sweden; 7 Department of Oncology-Pathology, Karolinska Institutet, Stockholm, Sweden; 8 Clinical Pathology and Cancer Diagnostics, Karolinska University Hospital, Stockholm, Sweden; 9 Clinical Genomics Stockholm, Science for Life Laboratory, School of Engineering Sciences in Chemistry, Biotechnology and Health, KTH Royal Institute of Technology, Stockholm, Sweden; 10 Clinical Genomics Stockholm, Science for Life Laboratory, Department of Microbiology, Tumor & Cell Biology, Karolinska Institutet, Stockholm, Sweden

**Keywords:** Hodgkin lymphoma, Translational research, Cancer genetics, Cancer genomics

Dear Editor,

Despite excellent cure rates in Hodgkin lymphoma (HL) [1], 10–15% of patients experience primary treatment failure or long-term treatment-associated complications due to overtreatment [2, 3]. Currently, interim positron emission tomography–computed tomography (PET/CT) with ^18^F-fluorodeoxyglucose, after two chemotherapy cycles, guides therapy in advanced stage-disease [4]. However, PET/CT has limited specificity, resulting in frequent false-positive and occasional false-negative findings [5]. underscoring the need for sensitive molecular tools for treatment response assessment and detection of measurable residual disease (MRD).

Longitudinal circulating tumor DNA (ctDNA) analyses enables sensitive MRD detection and dynamic treatment response monitoring in HL [6–9]. Several analytical approaches, primarily based on next-generation sequencing (NGS) technologies or droplet digital PCR (ddPCR), have been developed for sensitive ctDNA-based MRD detection. Targeted NGS-based MRD assays (NGS-MRD) employing unique molecular identifiers (UMIs) for error correction and statistical error-modeling of multiple baseline-informed reporter variants, including phased variants (PV), significantly enhance sensitivity and allow for dynamic MRD tracking [6]. We have previously demonstrated that multiplex ddPCR (m-ddPCR), tracking up to 3 reporter variants, allows cost-effective and sensitive longitudinal ctDNA-based MRD assessment [10, 11]. However, the comparative performance of these platforms for MRD detection in HL has not been evaluated.

Here, we report a head-to-head comparison of NGS-MRD and m-ddPCR for longitudinal ctDNA-based MRD detection in a population-based cohort of adults with HL enrolled in the BioLymph study (ISRCTN12948913) [12]. Tumor tissue-naïve baseline plasma genomic profiling was performed to inform assay design. We further assessed the prognostic value of ctDNA dynamics during primary treatment compared to PET/CT.

This study included a cohort of 36 patients with HL (Supplementary Tables S1–S2). Longitudinal plasma sampling was performed according to the BioLymph study protocol (Supplementary Table S3) [12]. Plasma samples were collected at baseline (Dx), after the first course (C1), at interim (start of cycle 3), at the end of primary treatment (EOT), during follow-up (1yFU, 2yFU), at relapse (R0) and during relapse treatment (R1, R2 etc.). PET/CT was performed as per clinical routine (Supplementary Table S3). Therefore, interim plasma was collected typically later than interim PET/CT (median 7 days interval, range -7 to 31). Most patients had advanced-stage disease (Stage IIB–IV; 63.9%), and the median age was 42 years (range, 19–84 years). Patients received first-line AVD/ABVD ± radiotherapy or escalated BEACOPP according to national guidelines. Patients were followed for a median of 59 months (range, 4–78 months); 5 relapses occurred at a median of 14.5 months (range, 6–40 months), including 2 cases of primary refractory disease.

Targeted sequencing was performed using 41.6 ± 10.7 ng (mean ± standard deviation) input cell-free DNA (cfDNA) from baseline plasma employing the Genomic Medicine Sweden Lymphoid gene panel [12]. with matched germline and normal plasma controls. Somatic alterations were identified in all patients at a mean target depth of 761 ± 149X unique duplex UMI-collapsed consensus reads (Fig. 1A, Supplementary Table S4). Detected alterations comprised single nucleotide variants (SNV) and indels with mean variant allele fractions ranging from 0.4% to 7.9%, including PV identified in 75% of cases, as well as recurrent copy-number variants (Fig. 1A, Supplementary Tables S4-S6). The recurrent mutational profile in baseline plasma from our patient cohort was consistent with prior HL studies [6, 7, 9].

**Fig. 1:**
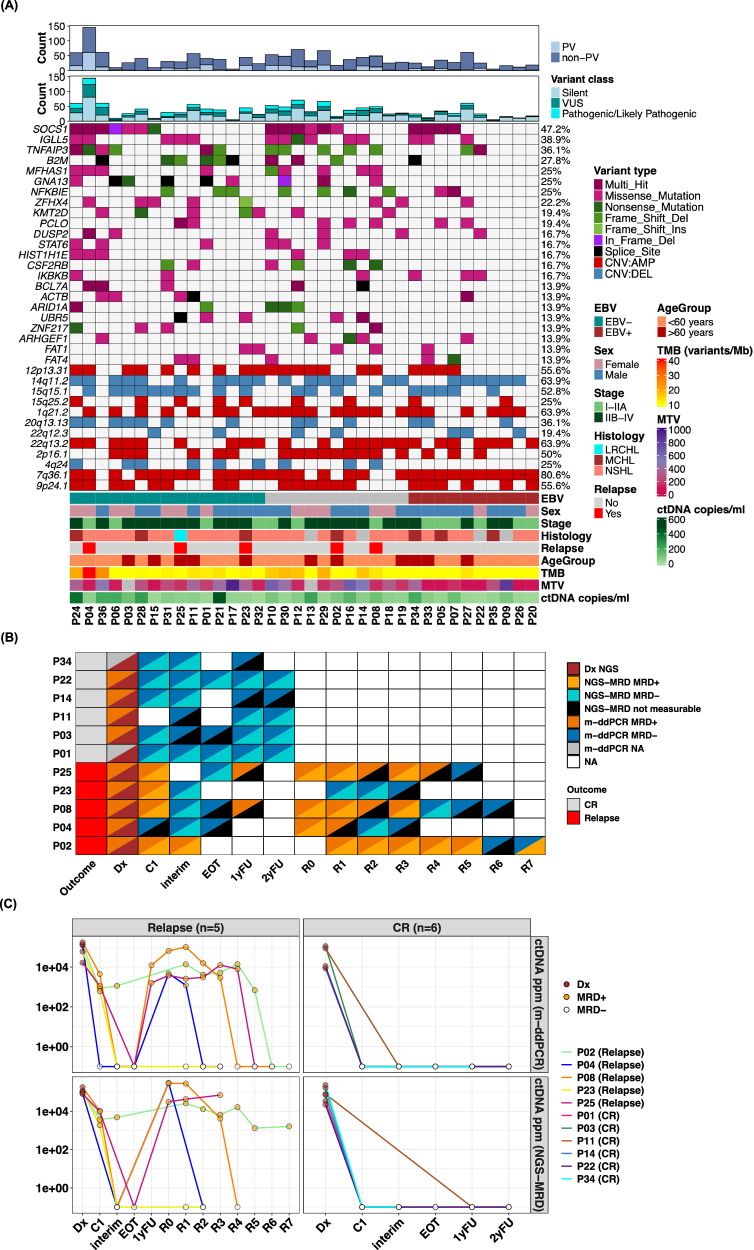
Evaluating m-ddPCR and NGS-MRD for baseline plasma-informed longitudinal monitoring in HL. **A** Somatic mutational landscape of HL profiled by deep targeted gene panel sequencing of plasma cfDNA collected at baseline in 36 patients. A total of of 1329 somatic SNV/Indels were detected across all patients. All patients had at least 1 non-synonymous SNV/Indel (range, 1–64), classified as either Pathogenic/Likely Pathogenic or variant of unknown significance (VUS). A total of 298 phased variants (PVs) were also detected across 27 (75%) patients. The oncoplot shows the recurrently altered genes with non-synonymous SNV/Indels and chromosomal arms with focal copy-number variants (profiled in all patients except P01 and P36), occurring in ≥5 cases alongside baseline clinical parameters, metabolic tumor volume (MTV), ctDNA concentration in copies/ml and outcome of the patients. **B** MRD detection by NGS-MRD and m-ddPCR compared in a sub-cohort of 5 cases with relapse and 6 cases in complete remission (CR), matched by age group, disease stage, and availability of plasma samples at matching timepoints. MRD status with both methods were compared in 66 follow-up samples. Baseline plasma (Dx) was also analysed by m-ddPCR when available (*n* = 9). NGS-MRD was not measurable in 21 follow-up samples that had <2 ng cfDNA amount. **C** Longitudinal kinetics of ctDNA molecules (in parts per million, ppm) measured by both NGS-MRD and m-ddPCR and stratified by clinical outcome. Pseudocount of 1 × 10^−1^ was added to all measurements for plotting on a logscale. VAF variant allele fraction, CNV copy-number variants, LRCHL lymphocyte-rich classical Hodgkin lymphoma, MCHL mixed cellularity classical Hodgkin lymphoma, NSHL nodular sclerosis Hodgkin lymphoma, TMB tumor mutational burden of non-synonymous variants/Mb of target panel region, AMP amplification, DEL deletion, CR complete remission, NA sample not available, Dx NGS baseline sample analysed by GMS-LGP, ppm parts per million, Dx diagnosis, C1 plasma sample after first chemotherapy course, EOT end-of-treatment plasma sample, 1yFU 1 year follow-up plasma sample, 2yFU 2 year follow-up plasma sample, R0 plasma sample at relapse, R1-R7 plasma samples collected after initiation of secondary treatment.

We compared the analytical performance of m-ddPCR and NGS-MRD for baseline plasma-informed targeted ctDNA analyses for longitudinal MRD assessment. For NGS-MRD, we employed a custom ~300 kb hybrid-capture MRD gene panel targeting both driver mutations and PV identified in baseline plasma, enabling MRD tracking in 35/36 patients (Supplementary Tables S7–S9, Supplementary Fig. S2). Libraries were prepared using a mean of 107 ± 98 ng cfDNA from 103 follow-up patient plasma samples. Deduplicated alignment files, consisting of unique single-stranded consensus reads were used as input for performing MRD assessment, applying Monte-Carlo simulation-based framework to evaluate detection of filtered reporter SNVs/PVs against background error rates specific to the reporter variant type. For direct comparison, MRD analyses were also performed by m-ddPCR in longitudinal plasma samples from a sub-cohort of 11 patients (5 relapsed/refractory cases and 6 matched cases in complete remission) using patient-specific m-ddPCR assays, targeting up to 3 reporter SNV/indels on the QX200 AutoDG Droplet Digital PCR System (Bio-Rad, Hercules, CA, US) as described previously [10, 11] (Fig. 1B, Supplementary Figs. S3–S4, Supplementary Tables S10–S11). As a novel approach, PVs were also incorporated as reporter variants in m-ddPCR assays for 3 patients.

Among the 66 longitudinal plasma samples from the sub-cohort used for the head-to-head evaluation of the two MRD platforms, 21 (32%) were below the technical input threshold for NGS-MRD ( < 2 ng of cfDNA), despite consistent plasma volumes available across analyses (Supplementary Fig. S5A). In contrast, m-ddPCR was successfully performed in all samples, demonstrating superior robustness in low-input settings. Notably, during follow-up, m-ddPCR detected ctDNA up to 6 months before clinical relapse in 2 cases with available plasma samples (Supplementary Fig. S6). A likely factor in low analyte levels in samples failing NGS-MRD was the longer delay between sampling and plasma preparation (median 91 h, range 3–143) compared to successfully analyzed samples (median 25 h, range 2–120) (Supplementary Fig. S5B).

In 45 follow-up samples analyzed by both NGS-MRD and m-ddPCR, MRD status was concordant in 44 samples (97.8%) (Fig. 1B). With both methods, ctDNA dynamics mirrored the clinical course. Increased or persistent ctDNA levels after EOT were observed in relapsed or primary refractory cases with samples available for analyses at follow-up or at relapse (*n* = 4), while MRD remained undetectable during follow-up in patients in remission (*n* = 6) (Fig. 1B–C). In concordant MRD-positive (MRD+) samples (*n* = 17), ctDNA molecules (in parts per million, ppm) detected by NGS-MRD were significantly higher than m-ddPCR (mean 65 530 ppm vs. 14 121 ppm, Wilcoxon signed-rank test *P* < 1.5 × 10^-5^) (Supplementary Fig. S7). This is consistent with the broader mutational coverage of NGS-MRD, and explains the single discordant sample deemed MRD+ by NGS-MRD only, owing to detection of a reporter variant not targeted by m-ddPCR (Supplementary Fig. S8). Subsequent imaging demonstrated progressive disease in this case; however, no further plasma samples were collected for analysis.

We next evaluated the prognostic relevance of MRD status and ctDNA kinetics during primary treatment in patients with ctDNA MRD assessment at C1 (*n* = 30), interim (*n* = 29), and EOT (*n* = 25) by NGS-MRD and/or m-ddPCR. At C1, 9/30 patients were  MRD+, of whom 4/9 (44.4%, 95% CI 13.7–78.8%) subsequently developed refractory disease or relapse, resulting in a significant association between MRD positivity at C1 and relapse (Fisher’s exact test, *P* < 0.019), which was not observed at interim (*P* < 0.261) (Fig. 2A). Importantly, 20/21 MRD- patients at C1 achieved complete remission, corresponding to a negative predictive value (NPV) of 95.2% (95% CI, 76.2–99.9%). Kaplan–Meier analysis confirmed significantly shorter progression-free survival in MRD+ patients (*P* < 0.049) compared to MRD- patients (Fig. 2B). In our patient cohort, we observed that <1.5 log_10_ fold-reduction in ctDNA concentration from baseline in samples analyzed by NGS-MRD (*n* = 29), distinguished relapsing patients (*n* = 4) from non-relapsing patients with residual MRD at C1 (*n* = 5), underscoring the value of quantitative ctDNA dynamics beyond binary MRD status (Fig. 2C). Alig et al*.* [6] observed a similar trend at the Cycle 1 Day 15 timepoint, corresponding to C1 in our study. While PV-restricted NGS-MRD approaches offer technical advantages to achieve higher sensitivity [6], tracking driver mutations in addition to PV, as performed in our study, provided a more biologically informative MRD assessment.

**Fig. 2:**
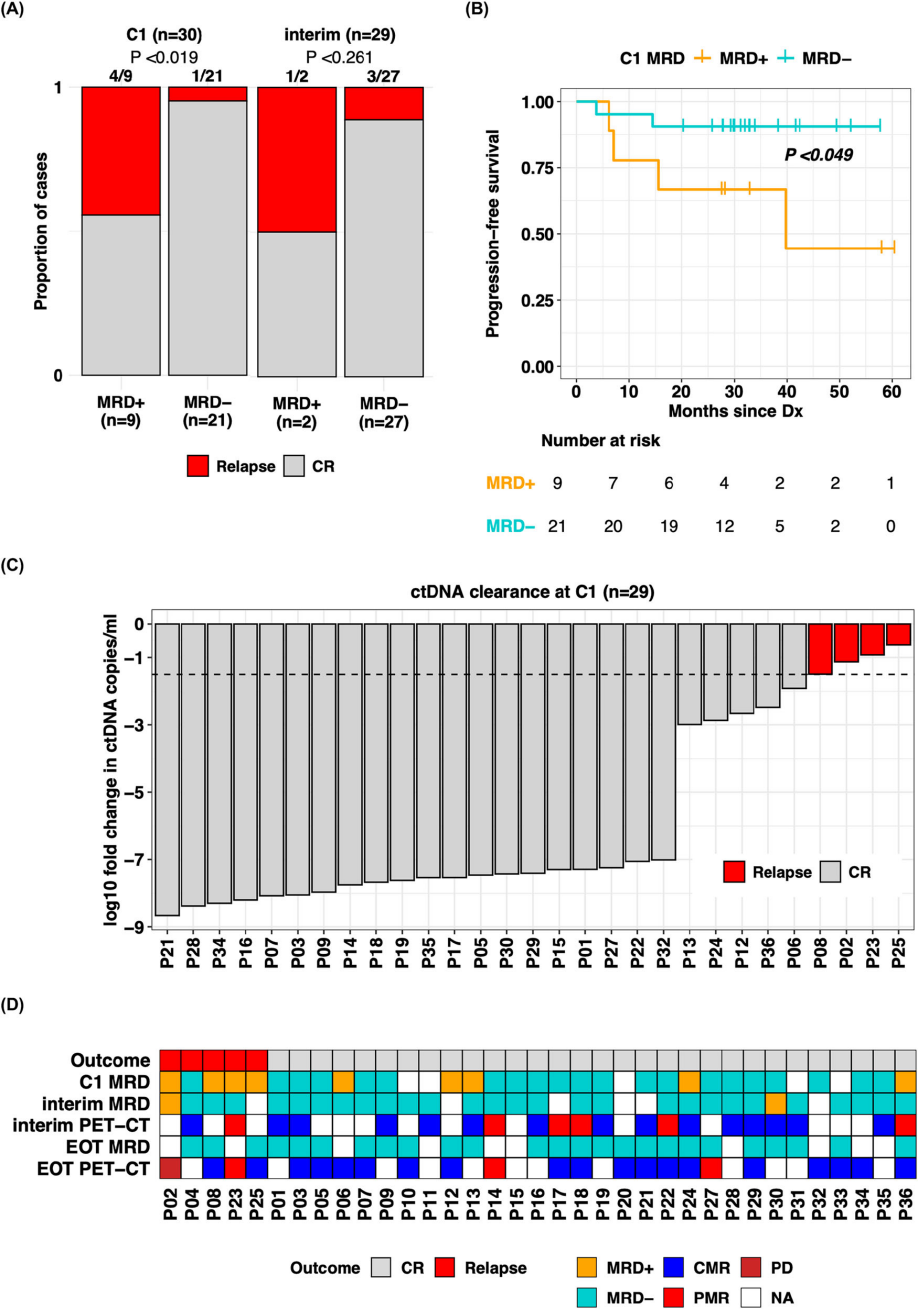
Predictive value of ctDNA-based MRD assessment during primary treatment. ctDNA-based MRD assessment during primary treatment performed in 35 patients using NGS-MRD and/or m-ddPCR. Plasma samples were available for 30 patients at C1, 29 patients at interim, and 25 patients at EOT. **A** Bar plot showing the proportion of cases stratified by outcome and MRD status at C1 and interim. Numbers above the bars indicate the number of cases that relapsed over the total number of patients within each subgroup. Distribution of outcomes between MRD+ and MRD- groups was tested using Fisher’s exact test. **B** Kaplan–Meier estimates of progression-free survival (PFS) according to ctDNA MRD status at C1, with the event defined as either relapse or death. Number of patients at risk in each group at different timepoints are shown in the table and significance between groups was tested using log-rank test. **C** Waterfall plot of ctDNA clearance at C1 analyzed by NGS-MRD (*n* = 29). Clearance of ctDNA levels was calculated as fold-change (in log_10_ scale) in ctDNA concentration at C1 relative to the baseline ctDNA concentration. A pseudocount of 1 × 10^-6^ was added to avoid undefined values for zero counts. **D** Comparison of interim and end-of-treatment (EOT) F-18 FDG PET/CT response evaluation and ctDNA-based MRD status, by NGS-MRD and/or m-ddPCR, at C1, interim and EOT, for predicting clinical relapse among 35 patients. At interim, F-18 FDG PET/CT data were available for 21 patients and ctDNA MRD results (by NGS-MRD and/or m-ddPCR) for 29 patients. At EOT, F-18 FDG PET/CT was performed in 22 patients and ctDNA MRD results were available for 25 patients. NA not available/analysed, CR complete clinical remission, CMR complete metabolic response, PR partial response, PD progressive disease, PMR partial metabolic response, C1 after first chemotherapy course, EOT end-of-treatment.

We then compared ctDNA-based response with PET/CT evaluations during primary treatment. At interim, only 1/6 PET/CT-positive patients relapsed (PPV 16.7%, 95% CI 0.4–64.1%), whereas 14/15 patients with a negative interim PET/CT achieved remission (NPV 93.3%, 95% CI 68.1–99.8%) (Fig. 2D). Among 27 MRD- patients at interim, 3 subsequently relapsed, resulting in a NPV of 88.9% (95% CI 70.8-97.6%). Concordance between PET/CT and MRD at interim was observed in 13/19 patients (68.4%, 95% CI 43.4–87.4%). Discordant results were predominantly represented by PET/CT-positive but MRD- patients (*n* = 5), of whom all but one remained relapse-free. At EOT, all 25 patients with available samples were MRD-, and apart from one patient, PET/CT and MRD were concordant. The discordant patient was PET/CT-positive but MRD- and received consolidative radiotherapy with no subsequent relapse.

We next compared the predictive value of ctDNA clearance at C1 with interim PET/CT response (Fig. 2D, Supplementary Fig. S9). Among patients with >1.5 log_10_-fold-reduction in ctDNA concentration who were PET-positive at interim (*n* = 5), none were refractory or experienced relapse. Despite the small cohort, MRD status at C1, with its high NPV, can therefore be a potential stratification biomarker, identifying PET-positive patients who might benefit from therapy de-escalation as shown in previous studies [6, 13–15].

To conclude, our study presents the real-world performance of ctDNA-based MRD detection in a consecutive population-based HL cohort. We demonstrate that longitudinal ctDNA monitoring was feasible by both m-ddPCR and NGS-MRD, providing highly concordant MRD assessments across multiple timepoints. Importantly, we show that ctDNA MRD evaluation provides significant prognostic information during primary treatment, most pronounced already at C1, and has the potential to refine early risk stratification, thereby supporting the implementation of personalized therapy in HL. Given the modest cohort size, these findings warrant validation in larger prospective cohorts.

## Supplementary information

Supplementary Methods

Supplementary Tables

Supplementary Figures

## Data Availability

The datasets presented in this article are not readily available because of identifiable sensitive patient data. Requests to access the datasets should be directed to the corresponding author.
