# Supplementary material for: Baseline plasma-informed circulating tumor DNA analyses comparing multiplex digital PCR and NGS for longitudinal monitoring in Hodgkin lymphoma
Source: Blood Cancer J. 2026 Jun 27;16(1):104. doi: 10.1038/s41408-026-01555-2 (PMC13310253; doi:10.1038/s41408-026-01555-2)
Supplement: Supplementary file 1 — Supplementary Methods [file 41408_2026_1555_MOESM1_ESM.pdf]

Supplementary Methods

Baseline plasma-informed circulating tumor DNA analyses comparing multiplex digital PCR and NGS for longitudinal monitoring in Hodgkin Lymphoma

Zahra Haider, Linn Deleskog Spångberg*, Karin E Smedby, Olha Krynina, Cecilia Jylhä, Irina Savitcheva, Emil Lundin, Marzia Palma, Lotta Hansson, Leonie Saft, Blaž Oder, Anna Lyander, Moa Hägglund, Anna Gellerbring, Mathias Johansson, Karl Nyrén, Richard Rosenquist, Tove Wästerlid* and Emma Tham*.*

**These authors contributed equally to this work*

Table of Contents

Patient cohort **3**

PET/CT evaluation **3**

Sample collection **4**

Plasma cfDNA extraction and quantification **4**

Genomic DNA extraction **5**

Sequencing Library preparation **5**

Pre-processing of gene panel sequencing data **6**

Targeted gene panel sequencing in baseline plasma samples **6**

Genomic profiling in baseline plasma **7**

- *i.* ***De novo variant calling using duplex consensus reads*** 7
- *ii.* ***Variant classification*** 7
- *iii.* ***Phased variants*** 8
- *iv.* ***Tumor mutational burden*** 8
- **Concentration of ctDNA copies in baseline plasma** 8
- **Copy number variant calling in baseline plasma** 9

Multiplex droplet digital PCR **9**

- *i.* ***Reporter selection and assay design*** 9
- *ii.* ***Multiplex droplet digital PCR analyses*** 9

NGS-MRD analyses in longitudinal plasma samples **11**

- *i.* ***MRD gene panel sequencing*** 11
- *ii.* ***Reporter variants*** 12
- *iii.* ***Reporter variant detection in follow-up samples*** 12
- *iv.* ***MRD estimation*** 14

Statistical analyses **14**

References **15**

Patient cohort

The present study initially included 43 patients with HL from the prospective BioLymph study cohort (ISRCTN12948913) (1). Patients received standard treatment of AVD (doxorubicin, vinblastine, dacarbazine)/ABVD (doxorubicin, bleomycin, vinblastine, dacarbazine) ± radiotherapy (RT) or escalated BEACOPP (bleomycin, etoposide, doxorubicin, cyclophosphamide, vincristine, procarbazine, prednisone, BEACOPPesc) based on age and Ann-Arbor staging as per Swedish National Guidelines. Early stage was defined as stage I-IIA and advanced stage as IIB-IV. EBV status was determined by EBER positivity in tissue. For this study, patients were excluded (n=7) if they lacked a baseline plasma sample collected at diagnosis (Dx) (n=2) and/or had no available sample at interim and EOT (n=5). Importantly, to enrich the cohort for relapses, all patients with relapsed or refractory disease (n=5) were included regardless of sampling completeness, resulting in a final study cohort of 36 patients (Supplementary Tables S1-2). The study was approved by the Stockholm Regional Ethical committee (2017/2538-31) and conducted in accordance with the Declaration of Helsinki. All participants provided written informed consent at inclusion.

PET/CT evaluation

PET/CT with ^{18}F-fluorodeoxyglucose (F-18 FDG) was performed according to clinical routine at diagnosis, and for a majority of cases, at interim (after two chemotherapy cycles), at EOT, and subsequently when clinically indicated (Supplementary Table S3). According to clinical guidelines, F-18 FDG PET/CT was not always required at interim, as patients with early-stage disease without risk factors could be evaluated clinically with or without low-dose CT, while those with early-stage disease with risk factors underwent interim CT. At EOT, F-18 FDG PET/CT could be omitted when the interim PET was negative. A full dose contrast enhanced CT was used at primary diagnostics and at EOT while low-dose CT was applied for interim control scans. All PET/CT scans were manually assessed by a senior consultant. Deauville score (DS) as well as other quantitative F-18 FDG PET/CT metrics were calculated including maximal standardized uptake value (SUVmax), total lesion glycolysis (TLG) and metabolic tumour volume (MTV). MTV evaluation was performed using Affinity Hermes Data Analysis Application v.3.0.4. Foci with pathological enhanced F-18 FDG uptake were defined with the support of CT-based characteristics. SUVmax of 2.5 was used as thresholds for delineation of

the hypermetabolic tumor lesions, and in cases where these lesions coalesced with areas of physiological uptake, active manual contouring was performed. The total volume of all hypermetabolic lesions was then merged into MTV. Both the PET/CT examinations and standalone CT scans were assessed according to the revised criteria for response assessment suggested by the Lugano classification (2).

Sample collection

For plasma preparation, blood samples from patients were collected in Cell-Free DNA BCT tubes (STRECK, La Vista, NE, US) and stored at room temperature for a maximum of 7 days before extraction. Samples were taken before the start of primary treatment (Dx), after the first chemotherapy course (C1) (corresponding to after course 1 for patients treated with BEACOPPesc and 1a for AVD/ABVD), at interim (corresponding to initiation of cycle 3 i.e. after course 2 in BEACOPPesc and after 2b for AVD/ABVD), and at EOT (1). Follow-up samples were collected once per year for 2 years post-diagnosis (1yFU, 2yFU), and in the event of relapse, before start of relapse treatment (R0) followed by during and after each course of relapse treatment (R1, R2, R3 etc.). Anonymized normal plasma samples from healthy donors were purchased from the blood bank. Peripheral blood from patients (n=36) and non-cancer patients (n=10) for matched germline control and normal genomic DNA (gDNA), respectively, was collected in BD Vacutainer® EDTA tubes (BD, Franklin Lakes, NJ, US).

Plasma cfDNA extraction and quantification

Cell-free plasma was extracted by double centrifugation; first at 1 600 ×g for 10 minutes at 4°C, followed by 3850 ×g for 10 minutes at 4°C. Plasma was aliquoted and stored at −80°C until further analyses. Plasma cfDNA was extracted using the QIAamp Circulating Nucleic Acid Kit™ (Qiagen, Hilden, Germany) on a QIAvac24 Plus vacuum manifold, according to the manufacturer's protocol. Plasma cfDNA was eluted either in 40 µl AVE buffer for ddPCR analyses or in 55 µl of nuclease-free water (NFW) for NGS.

Agilent Tapestation with the cell-free DNA ScreenTape assay (Agilent, Santa Clara, CA, US) was used to measure cfDNA concentration and assess cfDNA quality. High-quality cfDNA samples deemed "pass" for sequencing were characterized by dominant peaks at ~160–180 bp, with optional secondary peaks at ~320 bp and ~480 bp. Samples with genomic DNA contamination i.e. high-molecular-weight smear above 500 bp, were flagged. Concentration of cfDNA in plasma was presented in ng/ml by multiplying the concentration in eluted sample

with the ratio of elution volume (µl) and plasma volume (ml). Total cfDNA copies/ml in plasma was calculated by dividing concentration in ng/ml with the weight of 1 haploid DNA i.e. 0.0033 ng.

Genomic DNA extraction

Peripheral blood from patients (n=36) was collected in BD Vacutainer® EDTA tubes (BD, Franklin Lakes, NJ, US) and matching germline genomic DNA (gDNA) was extracted using the QIAsymphony system (Qiagen) according to the manufacturer's protocol and eluted in AVE buffer (Qiagen). For NGS library preparation, buffer exchange with elution in NFW was performed using Agencourt® AMPure® XP beads (Qiagen) according to the manufacturer's protocol for all patient gDNA samples.

Peripheral blood from non-cancer patients (n=10) was collected in BD Vacutainer® EDTA tubes (BD) and pooled, followed by extraction using QIAamp DNA Blood Maxi kit (Qiagen) according to the manufacturer's protocol. DNA was eluted in 1 ml buffer AE, followed by a second elution with 1 ml buffer to maximize gDNA yield. The eluates were pooled and stored at -20°C.

Sequencing Library preparation

Libraries were prepared with KAPA HyperPlus/Prep kit (Roche, Basel, Switzerland) with modifications described previously (3). Briefly, gDNA was enzymatically fragmented with an incubation time of 17.5 minutes. For adapter ligation, 0.55 µM (for input amounts of 25-250 ng) or 0.14 µM (for input amounts < 25 ng) of xGen® Duplex Seq adapters with 3 nucleotide long unique molecular identifiers (UMI) (Integrated DNA Technologies, IDT, Coralville, IA, US) were used. For PCR amplification, 2 mM xGen® Indexing primers with unique dual indices (IDT) were used with varying number of PCR cycles depending on the starting amount of input DNA; 5 cycles for 150-250 ng, 8 for 25-150 ng or 10 cycles for < 25 ng.

Target enrichment by hybrid-capture was performed following recommendations by Twist Biosciences (Twist Bioscience, San Francisco, CA, US) and as previously described (3). A total amount of 1500 ng of pooled indexed libraries were hybridized with probes of target capture panels with Twist Universal Blockers and Blocking solution. The hybridization mixture was incubated at 70°C for 18 hours. The post-capture PCR was performed with 0.5 mM xGen® Library Amp Primer (IDT) for 10 cycles. Pre- and post-hybridization libraries were quantified

using Quant-iT dsDNA HS assay (Invitrogen, Waltham, MA, US) and TapeStation HS D1000 assay (Agilent).

Pre-processing of gene panel sequencing data

Pre-processing of panel sequencing data was performed by BALSAMIC pipeline (4) where v.12.0.2 was employed for baseline patient samples, and v.16.0.0 for serially collected longitudinal samples, baseline patient samples for CNV calling and all normal cfDNA samples. Here, raw FASTQ files were first quality-controlled used using FastQC v.0.11.9. Adapter sequences and low-quality bases were trimmed using fastp v.0.23.2 (5). UMI tag extraction and consensus calling were performed using Sentieon® tools v.202010.02 (included in BALSAMIC v.12.0.2) or v.202308.03 (included in BALSAMIC v.16.0.0) (6). The alignment of UMI-extracted and consensus-called reads to the human reference genome hg19 was done by bwa-mem and samtools using Sentieon® utils.

Targeted gene panel sequencing in baseline plasma samples

Targeted gene panel sequencing was performed in baseline plasma cell-free DNA (cfDNA) with matched germline control gDNA and normal plasma cfDNA using the Genomic Medicine Sweden Lymphoid gene panel (GMS-LGP) (Twist Biosciences, San Francisco, CA, US) (1,7). The ~1.6 Mb panel, originally covering 252 genes and a genome-wide copy number backbone with probes distanced at 2 Mb as described previously (GMS-LGP v.7.2) (1), was updated during the study to include six additional genes (*IGLL5, RUNX1, PCLO, HLA-B, IL4R, BCR*) and increased backbone resolution with 1 Mb spacing between probes (GMS-LGP v.7.3). Two patients, P01 and P36, were analyzed with the GMS-LGP v.7.2, while the remaining patients and normal plasma samples were analyzed with the updated GMS-LGP v.7.3 panel.

For library preparation, a mean input amount of 41.6 ng ± 10.7 ng cfDNA from 4-5 ml patient plasma (n=36) was used, along with a mean of 28.3 ± 27.9 ng normal cfDNA (n=6) and 250 ng matched germline control gDNA (n=36). Baseline cfDNA and gDNA libraries were paired-end sequenced on a NovaSeq 6000 (Illumina, San Diego, CA, US) while normal cfDNA libraries were sequenced on a NovaSeq X (Illumina). Captured libraries were sequenced with a read length of 2x150 bp and aimed at generating 100 million read pairs (Mrp) per sample. Demultiplexing was performed using BCL Convert v.4.1.7 (Illumina).

Genomic profiling in baseline plasma

i. De novo variant calling using duplex consensus reads

For calling somatic single nucleotide variants (SNVs) and small insertions and deletions (indels), consensus reads were first filtered with Sentieon® based on default criteria of a minimum 3 reads supporting each UMI tag family and with at least one UMI tag existing in each single-stranded consensus read. The filtered duplex consensus reads were quality-controlled using Picard CollectHsMetrics v.2.27.1 (8).

Somatic mutations were called using Sentieon® TNscope® (9), with non-default parameters for passing the soft-filtered list of variants (–min_tumor_allele_frac 0.0005, –filter_t_alt_frac 0.0005, –min_init_tumor_lod 0.5, min_tumor_lod 4, –max_error_per_read 5 -pcr_indel_model NONE, GNOMADAF_popmax ≤2%). Variants were also annotated and filtered against loqusDB curated frequency of observed variants (frequency <1%) from non-cancer cases. All variants were annotated using Ensembl VEP v.104.3 (10) and vcfanno v.0.3.3 (11) for their population allele frequency from gnomAD v.2.1.1 (12), and SweGen (13) (Supplementary Figure S1).

Soft-filtered and annotated variants were hard-filtered as follows: >0.5% maximum observed allele frequency in 1000 Genomes, ESP and gnomAD ("max_AF"), >0.1% GNOMADAF, <200 total depth in plasma and <3 fold-difference of variant allele fraction (VAF) between matched normal gDNA and plasma cfDNA sample. Additionally, a panel of paired normals (POPN) was prepared by enlisting all variants called with a VAF >0 in any of the 36 matched normal gDNA samples. Variants occurring in ≥4 samples in the POPN or occurring in <4 samples in the POPN but with a ≤3 fold-difference between maximum VAF in POPN and minimum VAF in plasma sample were removed (Supplementary Figure S1). Variants were also filtered against a panel of normals (PON), comprising all loci with VAF >0 in 6 normal cfDNA samples. Additionally, variants occurring in pseudogenes or intergenic regions were removed.

ii. Variant classification

All variants with ClinVar annotation of "likely pathogenic", "pathogenic" or "uncertain significance" were rescued from the filtering steps and retained. None of these variants were implicated in the POPN-filter nor in the preceding population filters.

The resulting somatic variants were scored using GenomicScores R package with AlphaMissense (snapshot date 2024-04-30) and CADD Phred scores (cadd.v1.6.hg19,

snapshot date 2024-04-30) as well as analyzed with Molecular Tumor Board Portal (MTBP) v.7.3.4 (14), and SpliceAI (15). Variants with pathogenic, likely pathogenic and benign annotation in ClinVar were classified as such. Truncating frameshift, nonsense (e.g. start lost and stop gained) mutations, splice variants with SpliceAI delta score >0.5, missense variants in known hotspots or predicted as functionally relevant in MTBP or previously reported in COSMIC in hematological malignancies were classified as pathogenic/likely pathogenic variants. Missense variants predicted as damaging/deleterious/pathogenic by SIFT/Polyphen/AlphaMissense and with a CADD score ≥20 were also grouped with pathogenic/likely pathogenic variants. Missense variants reported in COSMIC but in other cancers or with a CADD score ≥20 were classified as variants of unknown significance (VUS).

Missense variants not reported previously, classified as functionally neutrally or benign by MTBP, predicted as tolerated/benign by SIFT/Polyphen/AlphaMissense, with a CADD score ≤10 or with low SpliceAI score (delta score<0.5) were classified as benign/likely benign. Variants occurring in 3' untranslated regions and intronic variants with low SpliceAI score were classified as synonymous/intronic variants. Together, synonymous/intronic and benign/likely benign variants were grouped together as silent variants. Synonymous/intronic and benign/likely benign variants occurring in more than 2 cases in the local database of cancer patients were termed as artefacts and removed.

iii. Phased variants

Phased variants (PVs) were defined as ≥2 somatic variants having the same phasing ID information (PID) included as part of the TNscope® haplotype-based variant calling output (9). A PV group was defined as the number of variants occurring in phase together i.e. having the same PID.

iv. Tumor mutational burden

Tumor mutational burden (TMB) was calculated as the number of non-synonymous variants per mega base (Mb) of the target region.

Concentration of ctDNA copies in baseline plasma

As the baseline cfDNA was not sequenced to saturation (mean duplication rate 63%), the concentration of circulating tumor DNA (ctDNA) (copies/ml) was estimated as a product of mean VAF of all non-synonymous variants and total cfDNA concentration in copies/ml.

Copy number variant calling in baseline plasma

Somatic copy number variants (CNV) were called using CNVkit v. 0.9.12 in baseline plasma samples analyzed with GMS-LGP v.7.3 (n=34). Panel sequencing data of patient plasma samples and 6 normal cfDNA samples was pre-processed with BALSAMIC v.16.0.0 and deduplicated single stranded UMI consensus sequences aligned to hg19 were used. Copy number reference was built by pooling alignments from 6 normal samples using default settings except for –drop-low-coverage, --min-mapq 30 and –split parameters enabled. Segments with copy number gains or losses were classified using an amplitude threshold of ≥0.02 and ≤–0.02, respectively.

For identifying significantly recurrent copy number and focal events, Gistic v.2.0 (16) was used on segment files with default settings except for the following parameters: amplification threshold (-ta) 0.02, deletion threshold (-td) 0.02, calculate significance by gene (-genegistic 1), include broad-level analysis (-broad 1), threshold for distinguishing between broad and focal events (-brlen) 0.98, confidence level to call the region containing a driver (-conf) 0.90, q-value significance threshold (-qvt) 0.05, perform with arm peel off (-armpeel 1) and gene collapse method (-gcm) extreme.

Multiplex droplet digital PCR

i. Reporter selection and assay design

Reporter target variant selection for patient-specific multiplex droplet digital PCR (m-ddPCR) assays was guided by the following criteria: high VAF, pathogenic/likely pathogenic variants over VUS, and PVs <20 bp apart. To further refine reporter target selection, the genomic sequence spanning ±150 bp around each reporter variant was manually inspected for GC/AT repeats and neighboring variants. Mutant and corresponding wild-type allele assays were designed via the Bio-Rad ddPCR Design Engine (Bio-Rad, Hercules, CA, US) and ordered with probes labelled with 5’-FAM™ or 5’-HEX™ fluorophores and 3’-Iowa Black® Fluorescent Quencher (Supplementary Table S10).

ii. Multiplex droplet digital PCR analyses

As previously described (7,17), ddPCR analysis was performed on the QX200 AutoDG Droplet Digital PCR System (Bio-Rad) according to manufacturer’s protocol. For singleplex ddPCR, a reaction mix of 22 µl was prepared with 1X ddPCR™ Supermix for Probes (No dUTP) (Bio-Rad), 1X FAM and HEX ddPCR assays and 11 µl of template DNA. For amplitude m-ddPCR, ddPCR

Multiplex Supermix (Bio-Rad) was used and different combinations of individual assay concentrations intended for multiplexing were tested and adjusted to an optimum ratio to ensure cluster definition between individual assays (Supplementary Table S10). Droplet generation was performed using QX200 AutoDG (Bio-Rad) followed by PCR amplification in SimpliAmp™ Thermal Cycler (Applied Biosystems, ThermoFisher Scientific, Waltham, MA, US) according to the following the program: denaturation at 95°C for 10 minutes, 40 cycles of 94°C for 30 s and annealing at optimum annealing temperature for 60 s, and 98°C for 10 minutes followed by an infinite hold at 4°C. The ramp rate was set at 2°C/second for all steps except at 1°C/second for the holding step. Optimization of m-ddPCR assays was carried out in accordance with Bio-Rad Rare Mutation Detection Best Practices Guidelines, using gBlocks™ or eBlocks™ Gene Fragments (IDT) as controls. Optimal annealing temperatures for each assay were determined by gradient ddPCR with testing range of 55°C - 65°C.

iii. MRD detection by m-ddPCR in serially collected plasma samples

MRD analyses was performed on longitudinally collected plasma cfDNA samples (n=66) from 11 patients using optimized, patient-specific m-ddPCR assays as described previously (7,17) (Supplementary Table S10). For each patient, 33 µl of cfDNA sample eluates from 2-5 ml of plasma were analyzed in triplicates with baseline plasma cfDNA when available, with corresponding gBlocks™ or eBlocks™ Gene Fragments (Integrated DNA Technologies, IDT, Coralville, IA, US) of each assay as a positive control, 3 wells of non-template control (NTC) and 12 wells of normal plasma cfDNA samples for false positive rate (FPR) detection. Data analyses was performed in the QX Manager v.2.0 Standard Edition software (Bio-Rad), where thresholds to define positive and negative clusters were manually set by visualizing 1D and 2D amplitude plots of control wells. Mutant and wild-type target copies were calculated as the product of concentration (copies/µl) in merged wells, number of replicate wells and 20 µl of reaction mix analyzed in a single well.

For each assay, variant allele fraction (VAF) of reporter targets in patient and control samples was retrieved from the Bio-Rad software. FPR for each assay was defined as the maximum VAF of the reporter target observed in normal plasma samples (Supplementary Table S10). The limit of detection (LOD) was estimated based on the total number of cfDNA copies analyzed per reaction, where, based on Poisson statistics, detecting ≥3 mutant copies correspond to a 95% probability of observing at least one positive droplet. A mean of 14 037

± 19 265 cfDNA copies from each follow-up plasma sample were analyzed by m-ddPCR, resulting in a mean LOD of 0.06% ± 0.05% (Supplementary Table S11).

For each sample, the average number of cfDNA copies analyzed across multiplexed assays and the total number of mutant copies detected from these assays, were summarized (Supplementary Table S11). Total ctDNA copies detected per sample were presented as parts per million (ppm) of average cfDNA copies in the sample. Patient cfDNA samples were called MRD-positive (MRD+) if the following criteria were fulfilled in merged wells: VAF of the reporter target was above the FPR of the target assay; the 95% confidence interval Poisson error bars of target concentration were non-overlapping between normal plasma and patient cfDNA sample; and at least three positive droplets were observed in total with at least 1 single positive droplet.

NGS-MRD analyses in longitudinal plasma samples

i. MRD gene panel sequencing

Longitudinally collected plasma cfDNA samples (n=103) from 35 patients and normal plasma samples (n=6) were sequenced after hybrid capture with a ~300 kb custom-designed MRD-panel (Twist Biosciences) (Supplementary Table S7). The custom-designed MRD panel (Twist Biosciences) targeted frequently mutated genes across lymphoma subtypes, *KRAS/NRAS/SF3B1* gene hotspots, regions enriched for PVs and was further supplemented with recurrently mutated genes from the patient cohort in this study, specifically targeting genes mutated in ≥2 patients with pathogenic/likely pathogenic variants with VAF ≥1%. The MRD-panel enabled baseline plasma-informed tracking of reporter variants in 35/36 patients, thereby excluding one patient (P26) from NGS-MRD analyses.

Libraries were prepared using a mean of 107 ng ± 98 ng cfDNA from follow-up patient plasma samples (2-5 ml) and a mean of 47 ng ± 45 ng cfDNA from normal plasma samples (Supplementary Table S9). Enriched libraries were paired-end sequenced on a NovaSeq X (Illumina) with a read length of 2x150bp and aimed at generating 60 Mrp per library. Demultiplexing was performed using BCL Convert v.4.1.7 (Illumina) and data was pre-processed using BALSAMIC v.16.0.0 (4). Deduplicated alignment files, consisting of unique single-stranded consensus reads aligned to reference genome hg19, were used as input for performing MRD assessment.

ii. Reporter variants

For each patient, a BED file of reporter variants, defined as SNVs and PVs profiled in baseline samples and overlapping with target region of the MRD-panel, was prepared. Reads harboring reference and alternate alleles of these reporter variants were counted in all corresponding follow-up plasma samples and normal plasma samples using Rsamtools::pileup v.2.22.0 in only primary mapped unique single-stranded consensus sequencing reads (scanBamFlag parameters: isDuplicate = F, isSecondaryAlignment = F) with minimum base quality 20 and minimum mapping quality 20. VAF was calculated as the number of reads with alternate alleles divided by the total depth at the variant position.

For MRD estimation, reporter variants were dusted to reduce false positive results. Reporter SNVs detected in ≥2 normal plasma samples were removed. Additionally, variants with stable VAF dynamics across baseline and follow-up samples were also filtered out as they could represent potential CHIP events or a second malignancy, as we hypothesized that reporter variants specific for monitoring response for HL therapy would have a more dynamic trajectory during treatment. To identify variants with stable VAF over time, VAF trajectories for each variant were evaluated across baseline and longitudinal samples of corresponding patient. For each variant, mean, standard deviation, and coefficient of variation (CV) across all time points, as well as the slope and p-value of a linear regression model fitted to VAF versus time were determined. Additionally, maximum absolute VAF change between time points was determined. Variants were classified as “stable” if they met the following criteria: CV < 10%, a non-significant regression slope (P >0.05) with a minimal slope magnitude, a maximum VAF deviation below 0.5% and no apparent trend shifts upon manual visual inspection of VAF trajectories. Additionally, all reporter variants in *TET2*, a gene reported to harbor higher frequency of CHIP variants were also filtered.

iii. Reporter variant detection in follow-up samples

Monte Carlo simulation-based framework (18–20) was employed to test the empirical significance of detection of the remaining reporter SNVs and PVs, against the corresponding sample-specific background error rate.

Sample-specific background error rate for the target region was determined using the function get_background_rate() from the ctDNAtools R package (21), with modifications. Here, Rsamtools::pileup was performed on alignment files using unique primary mapped single-stranded consensus reads of all samples, with minimum base quality 30 and minimum

mapping quality of 30. The sum of all mismatches detected were then divided by the total bases in the target MRD panel region. Mismatches with VAF >5% were considered as real mutations and therefore removed from background error rate estimation (parameter: vaf_threshold = 0.05). For reporter PVs, the individual SNVs in phase were merged as single events and tested against an adjusted background error rate as described previously (19,20). Briefly, using the merge_mutations_in_phase() function from the ctDNAtools R package, unique reads that carried alternate alleles of all individual SNVs in phase were retained and counted while reads with partial coverage of a PV group (likely artefacts) were ignored. The ratio of retained reads to total reads covering all the reporter SNVs in phase (purification probability) was then used to adjust the estimated background error rate (Supplementary Figure S9).

For testing the NULL hypothesis that a reporter variant was not significantly different from the background noise, Monte Carlo based sampling test was performed for each reporter variant with given sequencing depth at the position and random bionomial sampling of alternative allele reads under the background error rate, using the positivity_test() function of ctDNAtools R package. Binomial distribution was used in the simulation because it models the process of observing alternative allele reads among a fixed number of total reads (sequencing depth of the reporter mutation), assuming a certain mismatch error rate (background error rate). Monte Carlo simulations were iterated 10 000 times, and an empirical p-value was computed as the proportion of simulations where the simulated VAF was equal to or exceeded the observed VAF of the reporter variant.

The p-value threshold for significance detection of the variants was based on the p-value of the variant detection in the normal plasma samples. If the p-value of the variant detected in the MRD patient sample was <0.01 or was lower than the minimum p-value of the variant in normal plasma samples, the variant detection was considered above the background error rate in the tested plasma sample. Additionally, for reporter SNVs, significant detection required alternate-allele support on both forward and reverse reads while for PV reporters, support of ≥2 reads harboring the alternate alleles of all variants in phase was required. Also, VAF for significantly detected PVs was calculated as number of reads supporting alternate alleles of all variants in phase divided by the total number of reads spanning all variants in phase.

iv. MRD estimation

Follow-up plasma samples were classified as MRD-positive (MRD+) if at least 1 non-synonymous reporter variant was significantly detected based on the Monte Carlo simulation testing. Total number of molecules analyzed were determined as the average depth of unique single-stranded consensus sequencing reads across all reporter variants. For reporter PVs, only reads spanning all variants in phase were considered. In MRD+ samples, total number of mutant molecules detected were defined as the total number of unique single-stranded consensus sequencing reads with alternate alleles across significantly detected reporter SNVs and PV groups and was presented as parts per million (ppm) of the total molecules analyzed. Mean VAF in MRD+ samples was calculated as the average VAF of all significantly detected reporter SNVs and PV groups. For kinetic analyses of ctDNA burden across longitudinal samples, ctDNA concentrations in copies/ml were computed as a product of mean VAF and total cfDNA copies/ml in the sample. Fold change of ctDNA burden during primary treatment were measured against baseline ctDNA concentrations and presented in log2 scale.

Statistical analyses

Statistical analyses were performed using R (v.4.5.0). Linear correlations between continuous variables were tested using Spearman correlation test. Continuous parameters by discrete independent and dependent groups were tested using Wilcoxon rank-sum test or Wilcoxon signed-rank test, respectively. Associations between categorical variables were evaluated with Fisher's exact test. The Kaplan-Meier method was used to estimate progression-free survival, and differences between groups were compared using the log-rank test. Pvalues were two-sided and a value of ≤0.05 was considered statistically significant or otherwise indicated.

References

1. Cheson BD, Fisher RI, Barrington SF, Cavalli F, Schwartz LH, Zucca E, et al. Recommendations for Initial Evaluation, Staging, and Response Assessment of Hodgkin and Non-Hodgkin Lymphoma: The Lugano Classification. Journal of Clinical Oncology. 2014 Sep 20;32(27):3059–67.
2. Lyander A, Gellerbring A, Hägglund M, Elhami K, Wirta V. NGS method for parallel processing of high quality, damaged or fragmented input material using target enrichment. PLoS One. 2024 May 29;19(5):e0304411.
3. Foroughi-Asl H, Jeggari A, Maqbool K, Ivanchuk V, Elhami K, Wirta V. BALSAMIC: Bioinformatic Analysis pipeLine for SomAtic MutatIons in Cancer. 2021 Dec 20 [cited 2023 Feb 24]; Available from: https://zenodo.org/record/5794088
4. Chen S, Zhou Y, Chen Y, Gu J. fastp: an ultra-fast all-in-one FASTQ preprocessor. Bioinformatics. 2018 Sep 1;34(17):i884–90.
5. Freed D, Aldana R, Weber JA, Edwards JS. The Sentieon Genomics Tools - A fast and accurate solution to variant calling from next-generation sequence data. bioRxiv. 2017;
6. Haider Z, Wästerlid T, Spångberg LD, Rabbani L, Jylhä C, Thorvaldsdottir B, et al. Whole-genome informed circulating tumor DNA analysis by multiplex digital PCR for disease monitoring in B-cell lymphomas: a proof-of-concept study. Front Oncol [Internet]. 2023 Jun;13. Available from: https://doi.org/10.3389%2Ffonc.2023.1176698
7. Smedby KE, Wästerlid T, Tham E, Haider Z, Joelsson J, Thorvaldsdottir B, et al. The BioLymph study – implementing precision medicine approaches in lymphoma diagnostics, treatment and follow-up: feasibility and first results. Acta Oncol (Madr). 2023 Jun 3;62(6):560–4.
8. Li H, Handsaker B, Wysoker A, Fennell T, Ruan J, Homer N, et al. The Sequence Alignment/Map format and SAMtools. Bioinformatics. 2009 Aug 15;25(16):2078–9.
9. Freed D, Pan R, Aldana R. TNscope: Accurate Detection of Somatic Mutations with Haplotype-based Variant Candidate Detection and Machine Learning Filtering. bioRxiv [Internet]. 2018 [cited 2023 Feb 26]; Available from: https://doi.org/10.1101/250647
10. McLaren W, Gil L, Hunt SE, Riat HS, Ritchie GRS, Thormann A, et al. The Ensembl Variant Effect Predictor. Genome Biol. 2016 Dec 6;17(1):122.

11. Pedersen BS, Layer RM, Quinlan AR. Vcfanno: fast, flexible annotation of genetic variants. Genome Biol. 2016 Dec 1;17(1):118.
12. Karczewski KJ, Francioli LC, Tiao G, Cummings BB, Alföldi J, Wang Q, et al. The mutational constraint spectrum quantified from variation in 141,456 humans. Nature. 2020 May 28;581(7809):434–43.
13. Ameur A, Dahlberg J, Olason P, Vezzi F, Karlsson R, Martin M, et al. SweGen: a whole-genome data resource of genetic variability in a cross-section of the Swedish population. European Journal of Human Genetics. 2017 Nov 23;25(11):1253–60.
14. Tamborero D, Dienstmann R, Rachid MH, Boekel J, Lopez-Fernandez A, Jonsson M, et al. The Molecular Tumor Board Portal supports clinical decisions and automated reporting for precision oncology. Nat Cancer [Internet]. 2022 Feb;3(2):251–61. Available from: https://www.nature.com/articles/s43018-022-00332-x
15. Jaganathan K, Kyriazopoulou Panagiotopoulou S, McRae JF, Darbandi SF, Knowles D, Li YI, et al. Predicting Splicing from Primary Sequence with Deep Learning. Cell. 2019 Jan;176(3):535-548.e24.
16. Mayakonda A, Lin DC, Assenov Y, Plass C, Koeffler HP. Maftools: efficient and comprehensive analysis of somatic variants in cancer. Genome Res. 2018 Nov;28(11):1747–56.
17. Mermel CH, Schumacher SE, Hill B, Meyerson ML, Beroukhim R, Getz G. GISTIC2.0 facilitates sensitive and confident localization of the targets of focal somatic copy-number alteration in human cancers. Genome Biol. 2011;12(4):R41.
18. Wallander K, Haider Z, Jeggari A, Foroughi-Asl H, Gellerbring A, Lyander A, et al. Sensitive Detection of Cell-Free Tumour DNA Using Optimised Targeted Sequencing Can Predict Prognosis in Gastro-Oesophageal Cancer. Cancers (Basel) [Internet]. 2023 Feb;15(4):1160. Available from: https://doi.org/10.3390%2Fcancers15041160
19. Newman AM, Bratman S V, To J, Wynne JF, Eclov NCW, Modlin LA, et al. An ultrasensitive method for quantitating circulating tumor DNA with broad patient coverage. Nat Med. 2014 May 6;20(5):548–54.
20. Newman AM, Lovejoy AF, Klass DM, Kurtz DM, Chabon JJ, Scherer F, et al. Integrated digital error suppression for improved detection of circulating tumor DNA. Nat Biotechnol. 2016 May 28;34(5):547–55.

21. Alkodsi A, Meriranta L, Pasanen A, Leppä S. ctDNAtools: An R package to work with sequencing data of circulating tumor DNA. 2020.
22. Kurtz DM, Soo J, Co Ting Keh L, Alig S, Chabon JJ, Sworder BJ, et al. Enhanced detection of minimal residual disease by targeted sequencing of phased variants in circulating tumor DNA. Nat Biotechnol. 2021 Dec 22;39(12):1537–47.
23. Meriranta L, Alkodsi A, Pasanen A, Lepistö M, Mapar P, Blaker YN, et al. Molecular features encoded in the ctDNA reveal heterogeneity and predict outcome in high-risk aggressive B-cell lymphoma. Blood [Internet]. 2022 Mar;139(12):1863–77. Available from: https://ashpublications.org/blood/article/139/12/1863/483214/Molecular-features-encoded-in-the-ctDNA-reveal
