# Supplementary material for: Baseline plasma-informed circulating tumor DNA analyses comparing multiplex digital PCR and NGS for longitudinal monitoring in Hodgkin lymphoma
Source: Blood Cancer J. 2026 Jun 27;16(1):104. doi: 10.1038/s41408-026-01555-2 (PMC13310253; doi:10.1038/s41408-026-01555-2)
Supplement: Supplementary file 3 — Supplementary Figures [file 41408_2026_1555_MOESM3_ESM.pdf]

Supplementary Figures

Baseline plasma-informed circulating tumor DNA analyses comparing multiplex digital PCR and NGS for longitudinal monitoring in Hodgkin Lymphoma

Zahra Haider, Linn Deleskog Spångberg*, Karin E Smedby, Olha Krynina, Cecilia Jylhä, Irina Savitcheva, Emil Lundin, Marzia Palma, Lotta Hansson, Leonie Saft, Blaž Oder, Anna Lyander, Moa Hägglund, Anna Gellerbring, Mathias Johansson, Karl Nyrén, Richard Rosenquist, Tove Wästerlid* and Emma Tham*.*

**These authors contributed equally to this work*

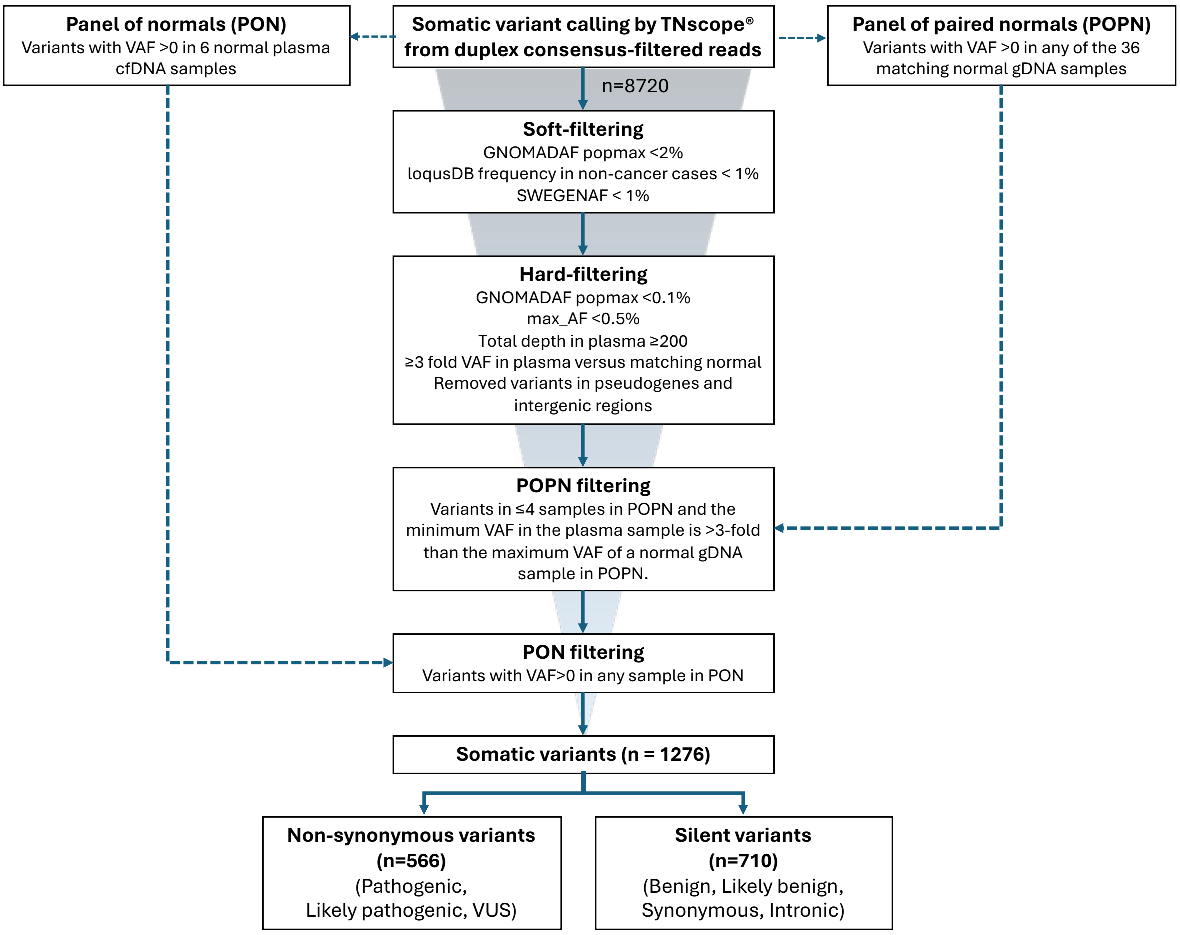

Supplementary Figure S1. Somatic variant filtering strategy for tumor-tissue naïve mutational profiling of baseline plasma ctDNA (n=36). For each patient, somatic variant calling, including single nucleotide variants (SNVs) and small insertions and deletions (indels), was performed by TNscope® in the Tumor-Normal mode using the matched germline control genomic DNA (gDNA) from whole blood. Variant calling was performed on alignment files with deduplicated UMI-collapsed duplex consensus reads. Panel of paired normals (POPN) constituted pooling together variant calls from all matching germline gDNA samples (n=36) from the patient cohort while panel of normals (PON) was composed of variant calls from normal plasma samples (n=6) from healthy donors.

VAF, variant allele fraction; gDNA, genomic DNA; max_AF, maximum observed allele fraction in 1000 Genomes, ESP and gnomAD

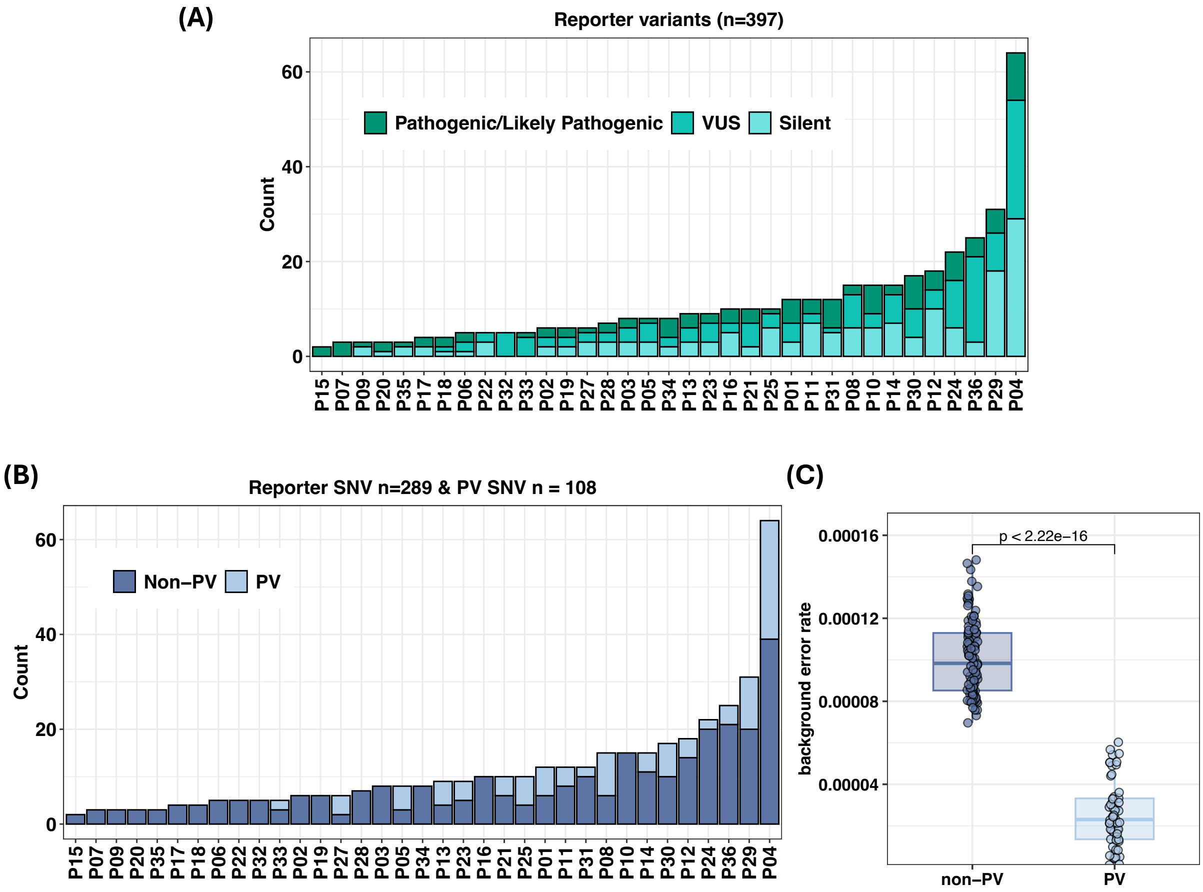

Supplementary Figure S2. Reporter variants and background error rate for NGS-MRD analyses across 35 cases. (A) A total of 397 reporter variants were used for MRD tracking by NGS-MRD across 35 cases. (B) Phased variants (PVs) consisting of ≥2 SNV in phase were also included as reporter variants in 18/35 cases. (C) Significant reduction of background error rate, tested by Wilcoxon rank sum test with continuity correction, was observed when employing PVs as reporter variants (mean $2.5 \times 10^{-5} \pm 1.6 \times 10^{-5}$) in longitudinal patient plasma samples (n=50) across 18 cases and normal plasma samples (n=6) compared to non-PV SNV as reporter variants (mean $9.9 \times 10^{-5} \pm 1.7 \times 10^{-5}$) in 103 longitudinal plasma samples across 35 cases and 6 normal plasma samples.

VUS, variants of unknown significance; SNV, single nucleotide variants.

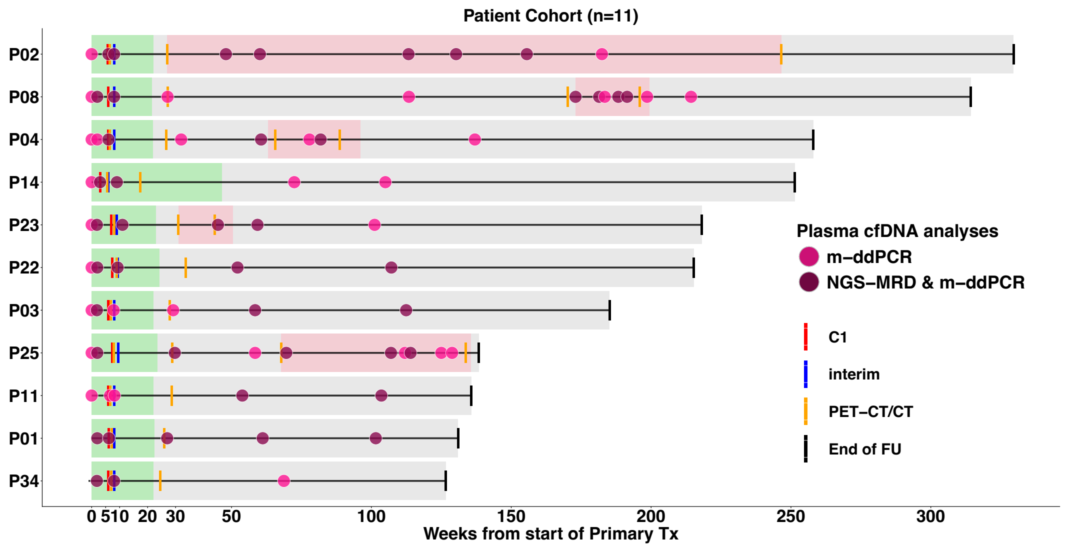

Supplementary Figure S3. Longitudinal plasma sampling and analysis in cases selected for comparison between NGS-MRD and m-ddPCR (n = 11).

Swimmer plot illustrating serial plasma collection (dots) in 11 patients (y-axis) evaluated for ctDNA-based MRD analyses using NGS-MRD and/or m-ddPCR. Diagnostic plasma samples were available in 9 patients for m-ddPCR analyses. For all cases, sampling and analyses methods are shown during primary treatment (green rectangle), C1, interim, EOT, and F-18 FDG PET-CT or stand-alone CT evaluation timepoints, as well as during follow-up. In 5 patients, plasma sampling during relapse treatment is also depicted (pink rectangle), beginning from start of secondary treatment to end of secondary treatment. *Primary Tx, Primary treatment; FU, follow-up; C1, after first chemotherapy course; EOT, end-of-treatment*

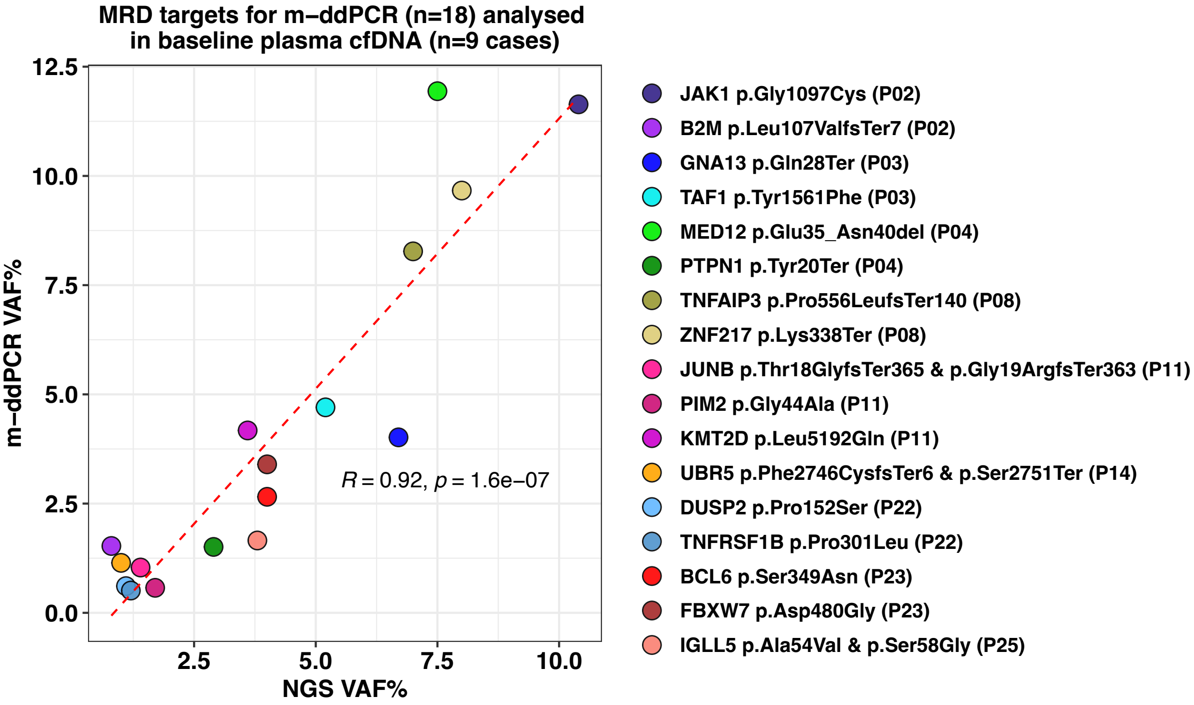

Supplementary Figure S4. Validation of m-ddPCR assays for reporter variant detection. Spearman correlation test of individual reporter variant allele fraction (VAF%) in baseline plasma cfDNA of 9 patients measured by patient-specific multiplex ddPCR assays (m-ddPCR), targeting 1-3 reporter variants per patient and baseline mutational profiling by targeted panel sequencing using GMS-LGP. The different reporter variants are presented with the corresponding patient in brackets.

(A)

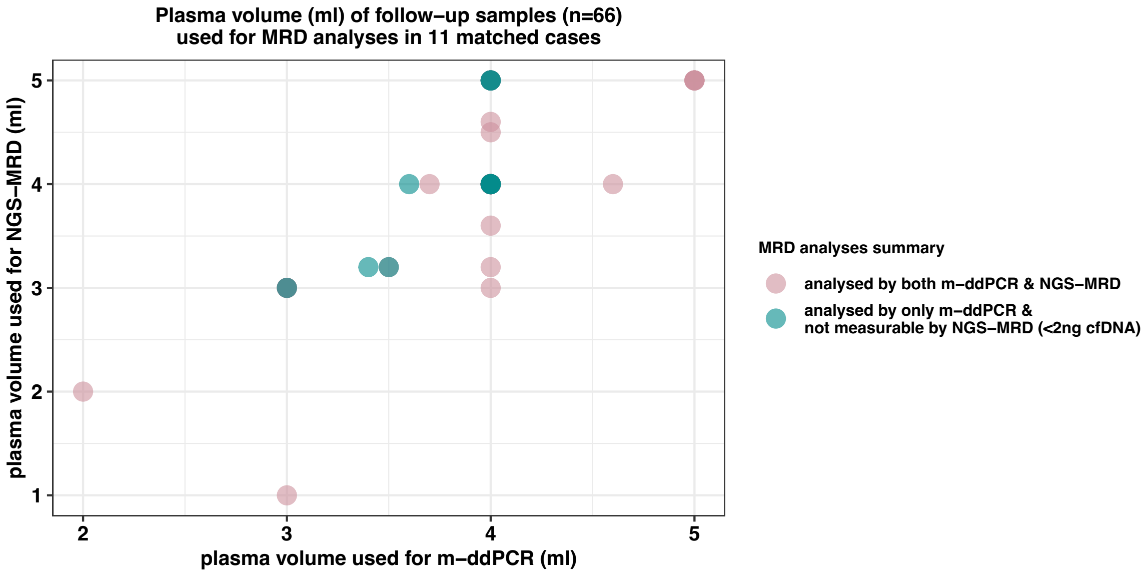

(B)

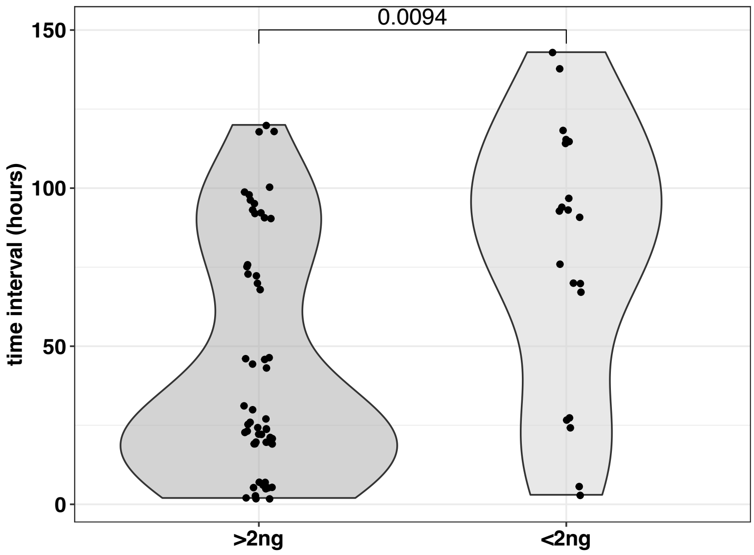

Supplementary Figure S5. Plasma sampling for NGS-MRD and m-ddPCR. (A) Comparable plasma volumes of longitudinal samples (n=66) from 11 patients were aliquoted for both NGS-MRD and m-ddPCR analyses. Samples not measurable by NGS-MRD, because of technical limit of assay for <2 ng of input cfDNA amount, had comparable plasma volumes with samples with >2ng cfDNA amount. (B) Mean time interval between sample collection and sample preparation was significantly shorter (tested by Wilcoxon rank-sum test) in samples with >2ng of cfDNA compared to samples that were not measurable by NGS-MRD owing to <2ng of input cfDNA.

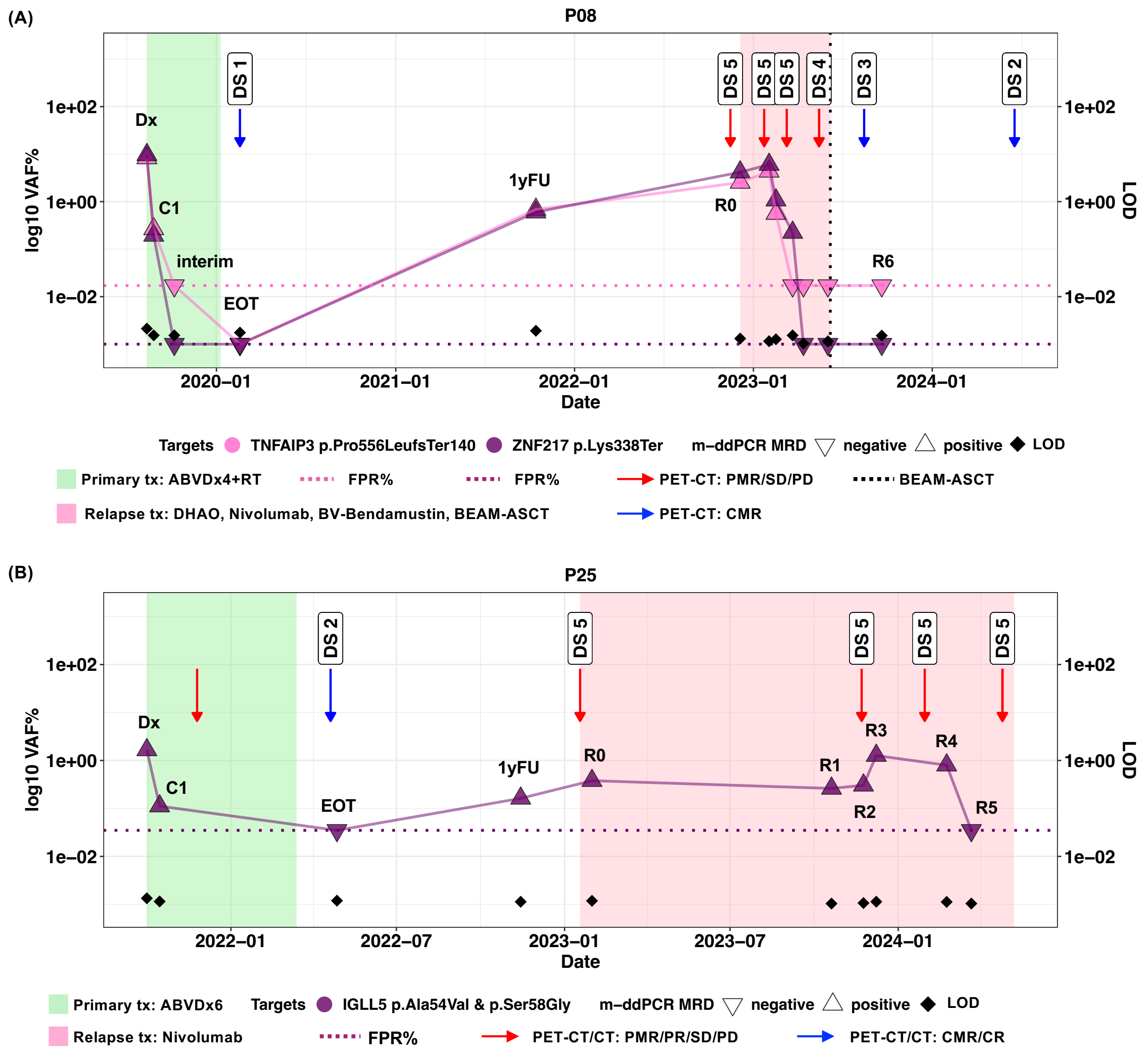

Supplementary Figure S6: Longitudinal ctDNA analyses for early detection of relapse. Longitudinal ctDNA dynamics measured by m-ddPCR across the complete clinical course of two patients: (A) P08 and (B) P25. In both cases, increase in plasma ctDNA levels preceded clinical manifestation of relapse. False positive rate (FPR) was determined as the maximum VAF measured by each mutant target assay in normal plasma samples. Pseudocount of 1 x 10^{-3} was added to all values, including limit of detection (LOD) (y-axis on the right), FPR (y-axis on the right) and VAF (y-axis on the right), for plotting on a log scale. For MRD- samples, VAF of the mutant target was replaced with the FPR of the target assay. Plasma ctDNA samples collected at 1-year follow-up (1yFU) after primary treatment (tx) were MRD+ for both patients. P08 relapsed 14 months after 1yFU sampling (A) while P25 relapsed two months after 1yFU sampling (B). Deauville score (DS) is provided when F-18 FDG PET/CT was performed. *BEAM–ASCT, carmustine (BCNU), etoposide, cytarabine, melphalan with autologous stem cell transplantation; ABVD, doxorubicin, bleomycin, vinblastine, dacarbazine; DHAO, dexamethasone, high-dose cytarabine (Ara-C), oxaliplatin; BV–bendamustine, brentuximab vedotin plus bendamustine; VAF, variant allele fraction; PET-CT, F-18 FDG PET/CT; CR, complete response; CMR, complete metabolic response; PR, partial response; SD, stable disease; PMR, partial metabolic response*

(A)

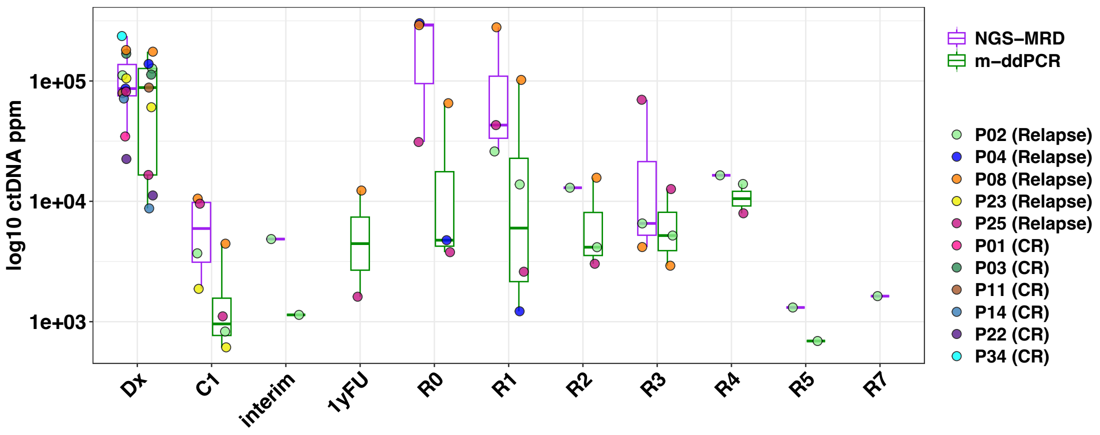

(B)

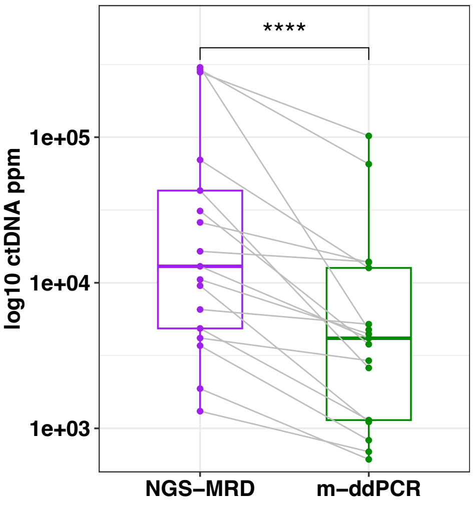

Supplementary Figure S7. (A) In follow-up samples deemed MRD+ from either method (n=24), ctDNA molecules detected, in parts per million (ppm), compared between NGS-MRD and m-ddPCR, and across different time points. (B) Comparison of ctDNA molecules detected, in parts per million (ppm) in follow-up plasma samples (n=17) concordantly deemed MRD+ by NGS-MRD and m-ddPCR. Statistical significance was tested by Wilcoxon signed rank exact test, with p-value <0.0001 indicated as ****.

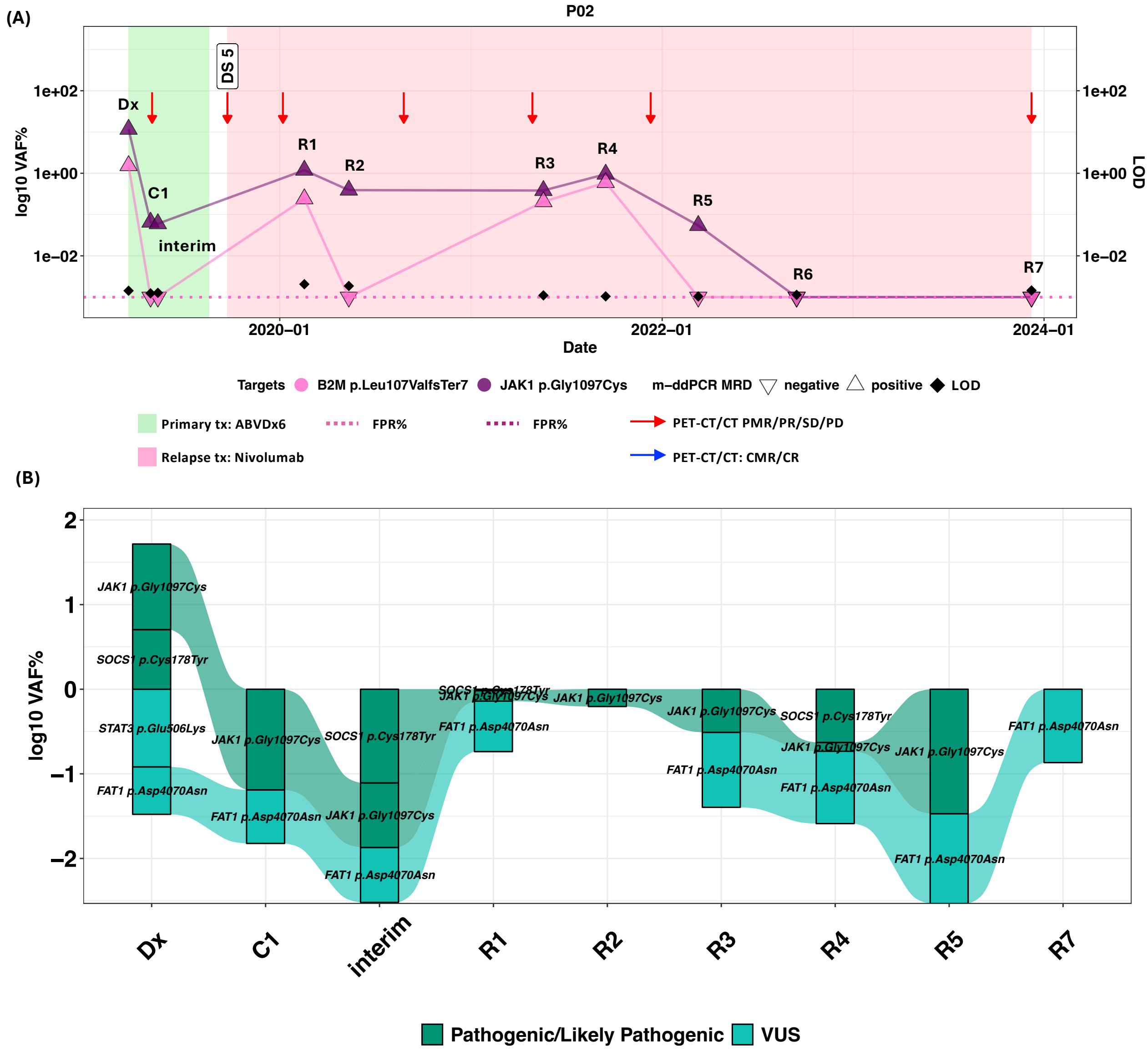

Supplementary Figure S8. Head-to-head comparison of MRD detection by m-ddPCR and NGS-MRD in patient P02. Longitudinal ctDNA-based MRD analyses spanning the clinical course of P02 by (A) m-ddPCR and (B) NGS-MRD. Two reporter variants were employed by m-ddPCR while NGS-MRD tracked 4 variants. *JAK1* p.Gly1097Cys was a common reporter variant concordantly detected (*or not detected*) by both methods. Shortly before R3, CT confirmed progression after two years off therapy, leading to Nivolumab discontinuation. The patient then received palliative radiotherapy with a short-lived response. Disease progression occurred again shortly after R4, prompting repeat radiotherapy, this time with a poorer response. Nivolumab was restarted prior to R5, and after R6 CT showed a good response; by R7 the patient achieved near-CR on CT, leading to treatment pause. However, here, NGS-MRD detected *FAT1* p.Asn4070Asn (0.02% VAF), rendering the sample MRD+, while m-ddPCR showed negative MRD. Eight months later, CT again confirmed progression, validating NGS-MRD evaluation at R7. *ABVD, doxorubicin/bleomycin/vinblastine/dacarbazine; CT, computed tomography; PET-CT, positron emission tomography–computed tomography; PR, partial response; CR, complete response; CMR, complete metabolic response; SD, stable disease; PD, progressive disease; LOD, limit of detection; FPR, false positive rate; VUS, variant of unknown significance; VAF, variant allele fraction*

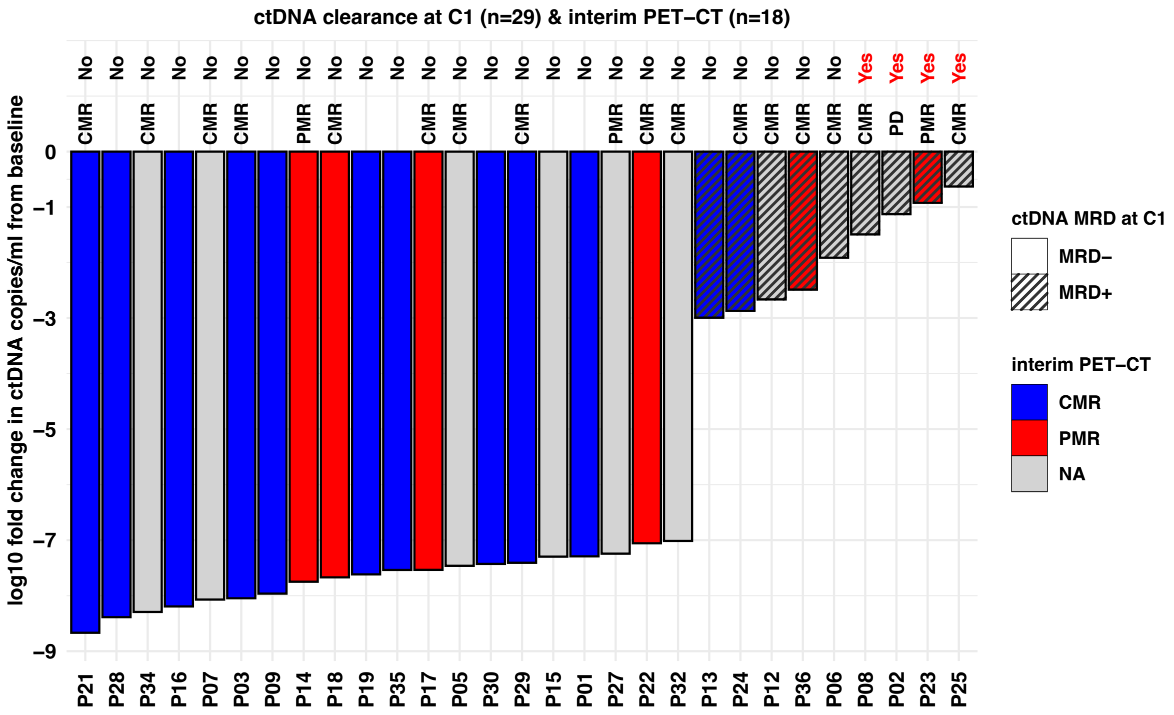

Supplementary Figure S9. Waterfall plot comparing predictive value of ctDNA clearance after first chemotherapy course (C1) (as $\log_{10}$ fold-change of ctDNA concentration from baseline) in 29 cases with F-18 FDG PET/CT evaluation at interim available for 18 of these 29 cases. A pseudocount of 1 x 10^{-6} was added to avoid undefined values for zero counts.

NA, not available/analysed; PET-CT, F-18 FDG PET/CT; CR, complete clinical remission; CMR, complete metabolic response; PR, partial response; PD, progressive disease; PMR, partial metabolic response; C1, after first chemotherapy course; EOT, end-of-treatment.
